# Effects of Alzheimer’s disease of varying severity on cardiac and autonomic function

**DOI:** 10.1590/1414-431X2021e11504

**Published:** 2022-01-05

**Authors:** Duyan Geng, Yan Wang, Zeyu Gao, Jiaxing Wang, Xuanyu Liu, Geng Pang

**Affiliations:** 1 State Key Laboratory of Reliability and Intelligence of Electrical Equipment, School of Electrical Engineering, Hebei University of Technology, Tianjin, China; 2 Key Laboratory of Electromagnetic Field and Electrical Apparatus Reliability of Hebei Province, School of Electrical Engineering, Hebei University of Technology, Tianjin, China

**Keywords:** Alzheimer’s disease, Cognitive ability, Electrocardiogram, Heart rate variability, Autonomic nerve function

## Abstract

Alzheimer’s disease (AD) is one of the most common neurodegenerative diseases in the elderly. The aim of this study was to explore the effects of AD on cardiac function and autonomic nervous function, and the feasibility of electrocardiogram (ECG) in monitoring the development of AD. APP/PS1 double transgenic mice were used in the Morris water maze (MWM) experiment to evaluate the changes of cognitive ability of AD mice, then the non-invasive ECG acquisition system was used and the changes of ECG intervals and heart rate variability (HRV) were analyzed. AD mice already had cognitive dysfunction at the age of 5 months, reaching the level of mild dementia, and the degree of dementia increased with the course of disease. There were no significant changes in ECG intervals in the AD group at each month. The mean square of successive RR interval differences, percentage of intervals >6 ms different from preceding interval, and normalized high frequency power component in the AD group were decreased and low-to-high frequency power ratio and normalized low frequency power component were increased. Combined with the results of the MWM, it was shown that the regulation mechanism of sympathetic and parasympathetic nerves in mice was already imbalanced in early stage AD, which was manifested as the increase of excessive activity of sympathetic nerves and the inhibition of parasympathetic activities. Therefore, ECG-based analysis of HRV may become a means of daily monitoring of AD and provide an auxiliary basis for clinical diagnosis.

## Introduction

Alzheimer’s disease (AD) is in one of the most common neurodegenerative diseases in the elderly, which has two pathological hallmarks. One is senile plaques formed by the excessive extracellular accumulation of β-amyloid (Aβ) protein and the other is the neurofibrillary tangles formed by the hyperphosphorylation of tau protein inside neurons (1). AD is mainly characterized by developmental memory decline, accompanied by declines in speech, behavior, and vision (2), which severely affect the quality of life of patients. Due to the hidden onset of AD, it is difficult to observe signs of the disease in its early stages, and when the symptoms are obvious, the patient’s condition is already serious. There is still no effective treatment for AD, but studies have shown that timely diagnosis and interventional therapy can help against the development of the disease (3).

Currently, there is no single detection method for AD, and doctors usually use multiple methods for diagnosis, including a detailed medical history and family medical history provided by the patient and family members, family opinions on the patient’s thinking skills and behavioral changes, cognitive testing combined with clinical examinations, etc. The clinical examinations include mostly blood tests, magnetic resonance imaging, cerebrospinal fluid biomarker detection, and positron emission computed tomography (4-7). However, the information provided by the family and the results of cognitive scales are highly subjective, resulting in a large incidence of error in AD diagnosis. In addition, most of the clinical exams are so expensive that utilization rate is low in community medical institutions that do not meet the needs of daily monitoring. Therefore, a simple, low-cost effective detection method is of great importance for determination of the development of AD.

An electrocardiogram (ECG) is a relatively simple, inexpensive, commonly used test. Heart rate variability (HRV) obtained from ECG signals refers to the changes in differences between successive ECG cycles, which can be used to quantitatively and noninvasively evaluate autonomic nervous function reliably, and can be obtained almost without the cooperation of patients with dementia (8). Low HRV in the elderly has been shown to predict overall cognitive decline better than cardiovascular or other diseases (9,10) and has been recommended as an early biomarker of cognitive decline (11). In parallel, the effect of AD on autonomic nervous function in humans has been established as “AD-related dysautonomia”, but it remains insufficiently characterized (12,13).

In this study, the APP/PS1 double transgenic mice, which were hybridized between PRP-HAPPK595N/M596L single transgenic dementia model mice and PRP-HPS1DE9 single transgenic dementia model mice, were used as the AD model. The Morris water maze (WMW) test was used to evaluate AD severity in mice at different months of age, and the ECG was obtained with a non-invasive method. The purpose was to study the changes of cardiac function and autonomic nerve function during AD progression, and to explore the feasibility of ECG monitoring of the progression of AD.

## Material and Methods

### Animals

The APP/PS1 double transgenic mice and C57BL/6J wild type (WT) mice were purchased from Beijing Huafukang Biotechnology Co., Ltd. (China). All mice were raised in an SPF animal laboratory with a photoperiod of 12-h light/dark and free access to water and food. The mice were divided into 5-, 7-, 10-, and 12-month-old groups, in which APP/PS1 mice were the AD group and C57BL/6J mice were the WT group for control, with 10 mice in each group. The procedures for this study were approved by the Biomedical Ethics Committee of Hebei University of Technology (report number: HEBUTaCUC2020003).

### Assessment of dementia severity

The MWM video analysis system (SA201, SANS, China) was used to assess the cognitive ability of AD mice. The system is composed of a circular pool, a mobile platform, and an image acquisition and processing system. The circular pool was divided into four quadrants: Northeast (NE), Northwest (NW), Southeast (SE), and Southwest (SW). Stickers of different shapes were attached to the center of the pool wall, slightly above the water surface, in each quadrant as position and direction references for the mouse. The platform was placed in the center of the NW quadrant and 1 cm below the water surface.

The experiment was carried out for 6 consecutive days. Each mouse was trained 4 times a day with an interval of 10 min between each test. All mice were placed in the SE quadrant for the first and fourth times, and randomly in the SW and NE quadrants for the second and third times. During each trial, mice were placed facing the wall of the pool and monitored for 60 s.

The index recorded in the experiment was the escape latency referring to the time from entering the water to landing on the platform. Mice that remained on the platform for 2 s were recorded as successful platform searches. After successful search, mice were left on the platform for 30 s to familiarize themselves with the platform. Mice that failed to find the platform within 60 s were recorded as escape latency of 60 s, and these mice were guided to the platform and left in the platform for 60 s. The average of four trainings per day was taken as the score for that day. The average escape latency of all mice for the 6 days in the WT group was the reference value, and the final score of each mouse in the AD group was the average score of 6 days. The percent difference between the final score of each mouse in the AD group and the reference value of the final score of the WT mice was calculated. When >20%, the mouse was considered demented, from 20 to 30% mild dementia, >30-40% moderate dementia, and >40% severe dementia.

### Electrocardiogram

ECGenie awake non-invasive ECG analysis system (ECGenie, Mouse Specifics, USA) was used to obtain the resting ECG of mice. The system included a data acquisition computer, acquisition platform, and signal amplifier. Mice were placed on the acquisition platform with feet in contact with the electrodes to form bipolar lead ECG. Then, the ECG signal was processed by the biological signal amplifier, so that the waveform of ECG changes could be displayed on the computer. ECG was recorded by LabChart software (version 8, AD Instruments Ltd., Australia). The sampling rate was 2 kHz.

### ECG parameters and HRV analysis

After the ECG, the e-MOUSE physiological waveform analysis software was used to analyze the collected data, and the ECG interval durations and HRV parameters of mice were obtained. The signals containing excessive noise or error waveform were pruned or removed. Stable ECG signals of the mice in the resting state were intercepted manually, and the data of mice whose resting heart rate could be affected by trauma (such as bleeding, bite marks, etc.) were excluded.

The ECG parameters of AD mice evaluated were heart rate (HR), PR interval duration, QRS complex duration, and QT dispersion (QTd). QTd is the difference between the longest QT and the shortest QT interval.

The analysis of HRV mainly includes time domain analysis and frequency domain analysis (14). Time domain analysis comprises mathematical calculations on the RR interval, and frequency domain analysis comprises the power spectrum of the signal. The time series signal of the RR interval is converted to the frequency domain by Fourier transformation and other methods. In the HRV time domain indicator, the square root of the mean of the squares of successive RR interval differences (RMSSD) and the percentage of intervals >6 ms different from the preceding interval (PNN6) were used because they reflect the intensity of parasympathetic nerve activity. The threshold of 6 ms used in adult mice was proportional to the 50 ms commonly used in human clinical studies (15,16). In the HRV frequency domain indicator, the ratio of low to high frequency power (LF/HF), normalized low frequency power component (LFn), and normalized high frequency power component (HFn) were used because they reflect the intensity of sympathetic nerve and parasympathetic nerve activities. The modulation balance between the two, among them LFn and HFn, could minimize the influence of the change of total power (TP) on the component values of low frequency power (LF) and high frequency power (HF) (17).

### Statistical analysis

Data are reported as means±SE. The *t*-test was used to compare the difference in escape latencies between the AD group and the WT group in the MWM test. One-way analysis of variance was used to analyze ECG parameters and HRV indexes in the multiple groups of the same strain, and *t*-test was used to analyze the differences between the AD group and the WT group at the same month of age. Statistical significance was set at P<0.05.

## Results

### Dementia assessment

In the MWM experiment, escape latency progression during training is a very important indicator (Table 1), and its length of time indicates the cognitive ability of mice. The shorter the time after several training sessions, the stronger the cognitive ability. On the first and second day of training, there was no significant difference in the escape latencies between groups, while from the third to the sixth day of training, the escape latencies of all AD groups were significantly longer compared with that of the WT group at same age.

**Table 1:** Mean escape latency of Alzheimer’s disease (AD) and wild type (WT) mice of different ages.

Day of training/Group	5 months old	7 months old	10 months old	12 months old
Day 1				
AD	50.22±5.25	54.44±6.56	56.73±2.67	59.29±1.22
WT	38.34±4.09	53.56±6.54	46.03±4.61	51.34±7.10
P	0.764	0.957	0.887	0.974
Day 2				
AD	38.89±4.47	38.23±9.68	58.27±1.73	55.74±1.56
WT	36.56±3.76	29.16±4.55	45.01±4.66	46.7±7.5
P	0.893	0.773	0.594	0.536
Day 3				
AD	27.73±4.48	35.96±6.9	43.07±6.24	54.19±6.88
WT	16.08±2.52	17.15±3.53	18.45±3.83	24.99±3.94
P	0.047*	0.044*	0.035*	0.028*
Day 4				
AD	29.85±4.32	30.43±3.68	36.55±6.33	43.55±1.19
WT	19.54±2.94	12.93±3.65	16.87±3.39	10.18±4.16
P	0.022*	0.017*	0.033*	0.014*
Day 5				
AD	17.56±2.73	24.13±4.77	26.43±6.00	38.98±4.19
WT	12.85±1.67	10.24±3.04	15.10±2.08	10.92±3.39
P	0.012*	<0.001**	0.025*	<0.001**
Day 6				
AD	17.92±3.83	24.84±3.34	28.54±5.84	35.83±6.98
WT	12.73±1.78	9.79±2.77	17.57±3.61	10.97±2.7
P	0.035*	0.027*	0.013*	<0.001**

Data are reported as means±SE (n=10/group). AD: APP/PS1 double transgenic mice; WT: C57BL/6J wild type mice. *P<0.05, **P<0.01, AD groups compared to WT groups at days 1 to 6 of training (*t*-test).

The reference values of 5-, 7-, 10-, and 12-month-old mice were calculated, and the values were 21.41, 22.14, 26.51, and 25.85 s, respectively. The percent difference between the final score and the reference value of each AD mouse was calculated, and results showed that the ratios of mice in the 5-month-old AD group were mainly distributed between 20 and 30%, the ratios of mice in the 7-month-old AD group were mainly distributed between 30 and 40%, and the ratios of mice in the 10- and 12-month-old AD groups were above 40%, indicating that the 5-month-old AD mice had mild dementia, 7-month-old AD mice had moderate dementia, and 10- and 12-month-old AD mice had severe dementia.

### Heart rate and ECG interval analysis

**Figure 1:**
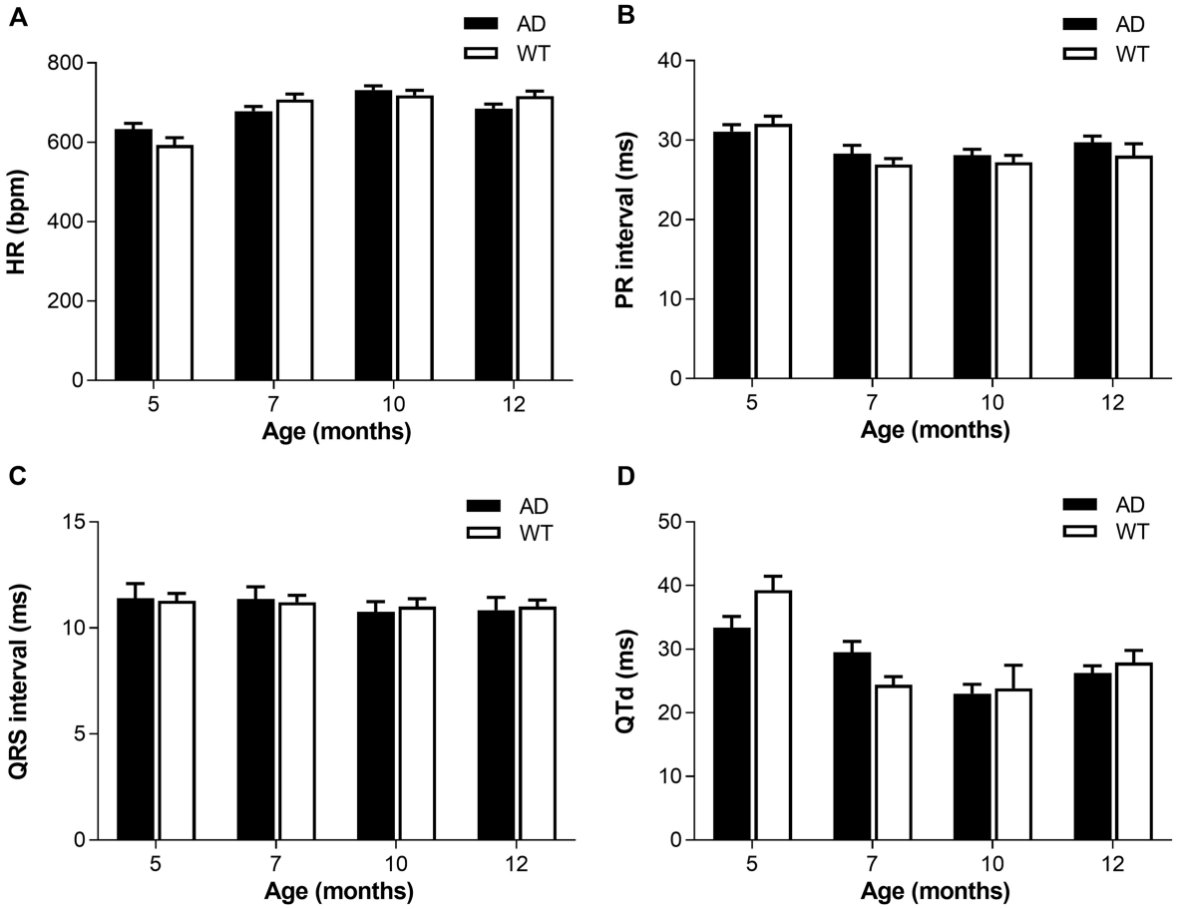
A, Heart rate (HR); **B**, PR interval; **C**, QRS interval duration; and **D**, QT dispersion (QTd) of Alzheimer’s disease (AD) and wild type (WT) mice of different ages. Data are reported as means±SE (n=10/group). AD: APP/PS1 double transgenic mice; WT: C57BL/6J wild type mice.

No difference was found in HR, PR interval duration, QRS interval duration, and QTd between AD and WT groups at any month of age (Figure 1A-D). Age had no significant effect on the duration of QRS interval duration (P>0.05). However, the differences in HR, PR interval, and QTd between groups at different months of age were statistically significant (P<0.05, one-way ANOVA). Compared with the WT group at 5 months of age, the HR of mice at other ages increased significantly, while the PR interval and QTd decreased significantly.

### Heart rate variability analysis

**Figure 2:**
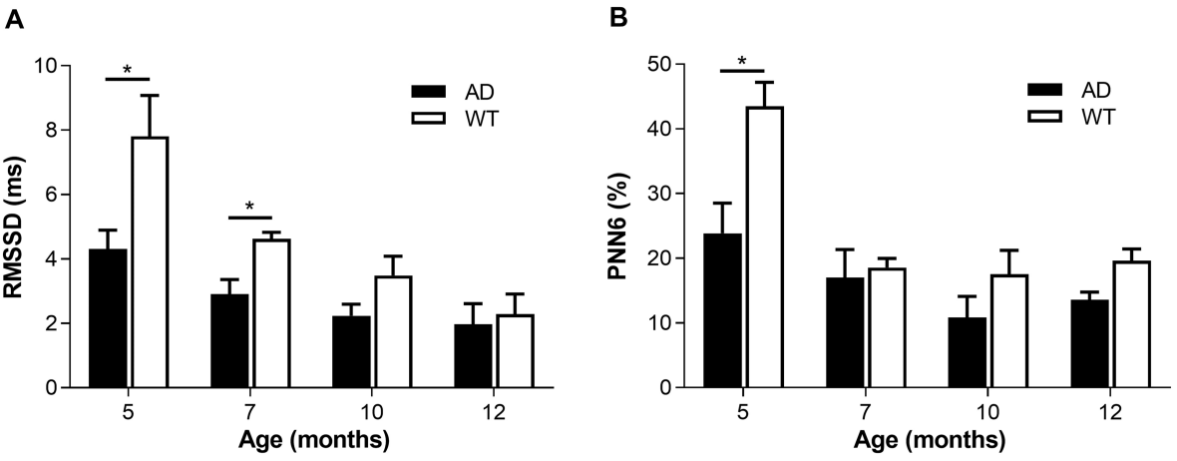
A, The square root of the mean of the squares of successive RR interval differences (RMSSD); **B**, percentage of intervals >6 ms different from preceding interval (PNN6) of Alzheimer’s disease (AD) and wild type (WT) mice of different ages. Data are reported as means±SE (n=10/group). AD: APP/PS1 double transgenic mice; WT: C57BL/6J wild type mice. *P<0.05 (*t*-test).

In the time domain indexes of HRV, both RMSSD and PNN6 reflect the intensity of parasympathetic activity. The effect of age on RMSSD and PNN6 was statistically significant (P<0.05). Compared with 5-month-old mice, RMSSD and PNN6 in 7-, 10-, and 12-month-old mice were significantly decreased (Figure 2A and B). Compared with the WT group, RMSSD and PNN6 in all AD groups decreased, and the RMSSD of 5-month-old and 7-month-old AD mice was significantly lower than that of the WT group at the same age (AD5=4.31±0.59 *vs* WT5=7.81±1.27 ms, AD7=2.91±0.45 *vs* WT7=4.63±0.20 ms; P<0.05), and the PNN6 in 5-month-old AD group was significantly decreased compared with the 5-month-old WT group (AD5=23.86±4.67 *vs* WT5=43.46±6.73 ms, P<0.05).

**Figure 3:**
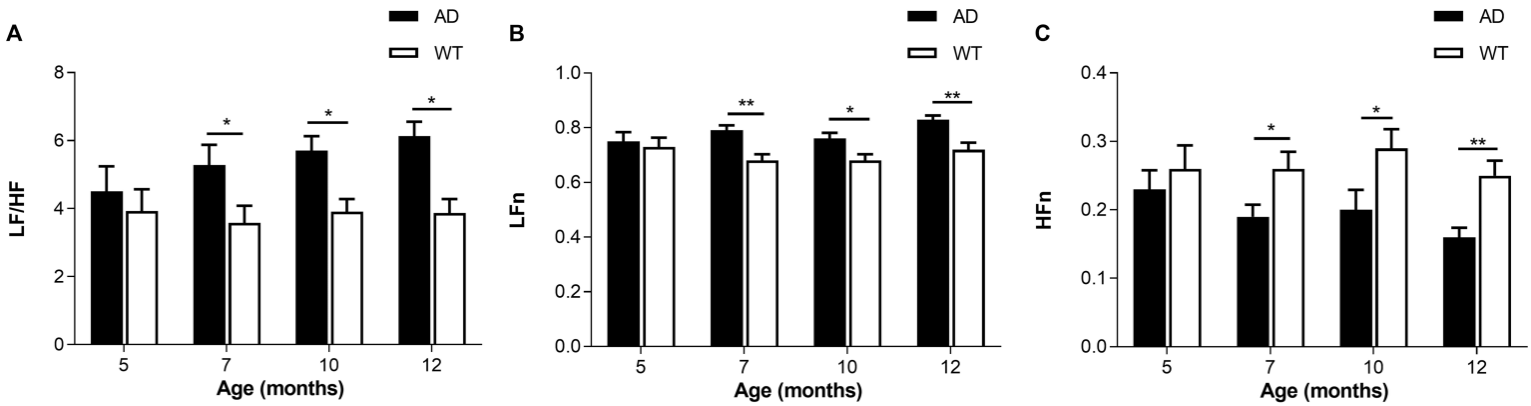
A, The ratio of low frequency power to high frequency power (LF/HF); **B**, normalized low frequency power component (LFn); and **C**, normalized high frequency power component (HFn) of Alzheimer’s disease (AD) and wild type (WT) mice of different ages. Data are reported as means±SE (n=10/group). AD: APP/PS1 double transgenic mice; WT: C57BL/6J wild type mice. *P<0.05; **P<0.01 (*t*-test).

In the frequency domain index of HRV, LF/HF reflects the modulation balance of sympathetic and parasympathetic nerves (Figure 3A), LFn reflects the activity intensity of sympathetic nerves (Figure 3B), and HFn reflects the activity intensity of parasympathetic nerves (Figure 3C). LF/HF and LFn in all AD groups were higher than those in the WT group, and HFn was lower than in the WT group. Moreover, the difference was statistically significant in 7-, 10-, and 12-month-old groups. Age had no significant effect on HRV frequency domain indexes of AD and WT groups (P>0.05).

## Discussion

In order to explore the level of condition in AD mice, the MWM experiment was used. Mice had mild dementia at the age of 5 months, moderate dementia at the age of 7 months, and severe dementia at the age of 10 and 12 months. The results were consistent with previous studies, which showed that the cognitive ability of APP/PS1 mice decreased gradually with the prolongation of AD course (18).

Protein misfolding plays a key role in the pathogenesis of neurodegenerative diseases, especially in AD (19). Past studies have suggested that AD has a common pathogenesis with heart disease. Mutations of presenilin 1 (*PSEN1*) and presenilin 2 (*PSEN2*) genes associated with familial AD have been found in patients with dilated cardiomyopathy (20). Furthermore, some studies have found Aβ aggregates, including Aβ40 and Aβ42, in myocardial cells and interstitial samples of AD patients, and abnormal ECG of AD patients, indicating that Aβ deposition caused by AD may damage cardiac function (21,22). That is why ECG parameters of AD mice at different ages were collected and analyzed in this study. In order to avoid the death of mice caused by improper injection of drugs during the collection of ECG in mice by traditional methods (23), the noninvasive awake ECG analysis system was used. The system has no restrictions on age and size of mice. It eliminates the interference that traditional methods have on the ECG of mice when using drugs, anesthesia, and human manipulation.

In order to explore the effect of AD on ECG parameters, the resting HR, PR interval, QRS complex duration, and QTd of mice were analyzed. HR is controlled by the sinoatrial node that is controlled by the autonomic nervous system and body fluid. HR is accelerated when a sympathetic nerve is excited and slowed down when the vagus nerve is excited. Our results showed that there was no significant difference in HR between the AD group and the WT group, but compared with 5-month-old mice, HR of 7-, 10-, and 12-month-old mice increased significantly, which indicated that sympathetic nerve activity increased the ageing of mice.

PR interval duration is an important indicator of heart conduction block and abnormal heart conduction, which reflects the time between atrial fibrillation and ventricular depolarization. The results of this study showed that compared with WT mice of same age, the duration of PR interval in AD mice of 7, 10, and 12 months of age was slightly prolonged, which indicated that there was a phenomenon of prolonged atrioventricular conduction in AD mice. There was no significant change in the QRS group reflecting the ventricular depolarization among each group. The difference between the longest QT interval and the shortest QT interval is called QTd. In past studies, the QTd was considered an indicator of uneven ventricular repolarization, and a significant increase in QTd represented a deepening of the disorder at the ventricular repolarity, which was associated with an increased risk of cardiovascular disease and was a sign of sudden cardiac death and ventricular fibrillation (24). High QTd was associated with autonomic dysfunction and other cardiovascular diseases (25-28). QTd of elderly people with cognitive impairment is higher than that of healthy people and is related to the degree of cognitive impairment (29). The results showed that there was no significant difference in QTd between AD and WT groups, and there was no obvious trend of change, which may be due to the small sample size. In the study of ECG parameters, age had a significant effect on HR, PR interval, and QTd, which highlighted the importance of age-matching the control mice.

The autonomic nervous system mainly includes sympathetic nerves and parasympathetic nerves, which jointly regulate body balance. Autonomic nerve function and cognitive function are closely related (30), and previous studies have found that in the early stage of AD, the autonomic nerve function of patients is impaired even before obvious symptoms (31,32). However, the relationship between HRV and autonomic dysfunction in patients with dementia is still controversial. Mellingsæter et al. (33) showed that patients with AD and mild cognitive impairment have poor sympathetic response to postural stress. Issac et al. (34) found that AD patients have dominance of sympathetic activity, while parasympathetic nerves are inhibited. Santos et al. (35) found that parasympathetic activity is inhibited in Aβ-positive patients under cognitive pressure. The results of the current study showed that there was a significant difference in RMSSD between the AD group and the WT group when the cognitive ability of mice was impaired at 5 months of age. With an increase in age, the LF/HF of AD mice also showed a trend of sustained growth, and there were significant differences between AD and WT groups at 7, 10, and 12 months of age. The LF/HF and LFn of AD mice were higher than those of normal mice, indicating that the regulation mechanism of sympathetic and parasympathetic nerves in AD mice was unbalanced, and the sympathetic activity was relatively high. HFn and RMSSD of AD mice were lower than normal mice in reflecting parasympathetic nerve function, indicating that AD can inhibit parasympathetic nerve activity in mice. Higher parasympathetic nerve activity is associated to reduced mortality by cardiovascular diseases and overall diseases, while excessive sympathetic activity will lead to reduced cerebral blood flow (34), which may explain the high mortality of AD patients in intermediate and late stages.

In conclusion, the autonomic nervous function of mice in the early stages of AD was abnormal, showing that the sympathetic overactivity was enhanced and the parasympathetic activity was inhibited. This is reflected in the HRV parameters that change with the development of the disease course. Therefore, it can be speculated that HRV may be a non-invasive physiological marker for early understanding of AD severity, but further studies are required to reveal the effect and mechanism of HRV on the changes of AD severity of patients.
